# Fibroblast growth factor receptor inhibitors in glioma: a narrative review of recent advances

**DOI:** 10.3389/fphar.2025.1714696

**Published:** 2026-01-06

**Authors:** Mingyu Han, Zhaokai Zhou, Bi Qian, Yuanqi Zhang, Cheng Peng, Fu Peng

**Affiliations:** 1 West China School of Pharmacy, Sichuan University, Chengdu, China; 2 Key Laboratory of Standardization of Chinese Medicine (Chengdu University of Traditional Chinese Medicine), Ministry of Education, Chengdu, China; 3 Department of Urology, The Second Xiangya Hospital of Central South University, Changsha, Hunan, China; 4 Department of Radiation Oncology, Peking Union Medical College Hospital, Chinese Academy of Medical Sciences and Peking Union Medical College, Beijing, China; 5 Affiliated Hospital of Guangdong Medical University, Guangdong Medical University, Guangzhou, China; 6 Key Laboratory of Drug-Targeting and Drug Delivery System of the Education Ministry, Sichuan Engineering Laboratory for Plant-Sourced Drug and Sichuan Research Center for Drug Precision Industrial Technology, Sichuan University, Chengdu, China

**Keywords:** glioma, fibroblast growth factor receptor (FGFR), pathways, gene fusion, inhibitors

## Abstract

Gliomas are devastating CNS malignancies characterized by extreme molecular heterogeneity and poor prognosis; the fibroblast growth factor receptor (FGFR) signaling axis, which drives proliferation, stemness, and metabolic adaptation, has thus emerged as a crucial therapeutic target. This review systematically synthesizes recent advances in understanding FGFR dysregulation, the clinical application of FGFR inhibitors, and the overriding pharmacological hurdles to achieving effective CNS exposure. FGFR signaling is dysregulated in gliomas by a range of genomic alterations, including mutations, amplifications, and key oncogenic fusions (e.g., FGFR3-TACC3). Moreover, contemporary investigations have demonstrated that novel structural changes in FGFR2 and FGFR3 are frequently linked to an aggressive tumor biology and specific gene expression signatures, thus validating their function as powerful, clinically actionable drivers. Pharmacologically, dedicated inhibitors like Infigratinib have demonstrated anti-tumor activity in clinical Phase II trials for FGFR-altered recurrent gliomas, while the multi-kinase inhibitor Regorafenib has shown a modest survival benefit in recurrent GBM; however, mechanistic studies indicate that effective response often relies on co-targeting bypass pathways (e.g., CLK2) and mitigating the tumor’s metabolic dependency. Crucially, limited drug exposure through the blood-brain barrier (BBB) continues to be the foremost challenge, dictating optimization efforts toward compounds with favorable pharmacokinetic properties and novel delivery platforms, such as the covalent inhibitor futibatinib and liposomal formulations, to enhance brain penetrance. In conclusion, the evolving molecular landscape validates FGFR alterations as a targetable niche in gliomas, and future success depends critically on integrating comprehensive next-generation sequencing to identify aggressive FGFR variants, developing next-generation inhibitors with superior BBB permeability, and implementing rational combination strategies to achieve durable clinical benefit.

## 1 Introduction

Brain tumours represent a significant clinical challenge due to their inherent local aggressiveness, resulting in high morbidity and mortality (Xu et al., 2025). A significant proportion of tumorous lesions within the brain are metastases, which arise from malignancies located outside the central nervous system (Gavrilovic and Posner, 2005; Chen et al., 2022; Luo et al., 2020). Conversely, gliomas and meningiomas are recognized as the two most common categories of tumors that originate directly in the brain (Ferlay et al., 2010; Ostrom et al., 2014). Accounting for approximately 30% of all primary CNS neoplasms and a substantial 80% of those deemed malignant, gliomas are the dominant fatal diagnosis in the primary brain tumor patient population. The cellular ancestry of these varied tumors is believed to be traced back to glial stem cells or their respective progenitor cells (Wang et al., 2018). Historically, gliomas were classified based on their morphological similarity to glial cells (e.g., astrocytoma, oligodendroglioma), with the malignancy grade (WHO 1–4) determined by the presence of mesenchymal features such as high mitotic activity and necrosis. Clinically, common adult gliomas include different grades of infiltrating astrocytomas, most notably Glioblastoma (GBM, WHO Grade 4), while pediatric gliomas are frequently pilocytic astrocytomas and diffuse midline gliomas (Weller et al., 2015).

The main causes of gliomas are the following: 1). Genetic predisposition; 2). Ionising radiation; 3). Non-ionising radiation; 4). Immune function (including allergies and infections); 5). Identified neurocarcinogens; and 6). Risk factors such as metals (Bondy et al., 2008). The pathophysiological mechanisms that give rise to gliomas are similar to those of other tumours, which are essentially single-cell genetic disorders (Table 1), for example, mutations in IDH, TP53, and deletions in PTEN, especially in glioblastoma (GBM), have a relatively low rate of IDH mutation, but are closely related to prognosis (Eckel-Passow et al., 2015; Chen et al., 2017; Illana et al., 2025; Bai et al., 2025; Cancer Genome Atlas Research Network et al., 2015; Chen et al., 2019; Aebisher et al., 2024). Although IDH mutation rates are relatively low in GBM, these molecular features are now crucial for classification and are closely tied to patient prognosis. Given the limited efficacy of conventional therapies, global attention has increasingly focused on improving treatment strategies, which currently include immunotherapy, targeted therapy, and electric field therapy. Substantial progress in elucidating the molecular pathogenesis of gliomas has not only refined diagnostic and classification systems but has also successfully identified novel molecular targets, outlining promising strategies for future prognostic improvement. Among the investigative pathways of interest are the Fibroblast Growth Factor Receptors (FGFRs). This collective of receptor tyrosine kinases (RTKs) is indispensable for controlling fundamental biological phenomena, specifically cell division (proliferation), cellular specialization (differentiation), and viability (survival) (Xue et al., 2018; Babina and Turner, 2017; Zhang et al., 2024). Comprising four paralogs (FGFR1-4), the FGFR family initiates a range of downstream signaling events. Specifically, individual FGFR members are capable of activating various signaling axes, such as the well-established PI3K/AKT and MAPK/ERK cascades (Tripathi et al., 2023; Wu et al., 2022). These pathways are critical for cellular homeostasis, and their dysregulation is frequently observed in cancer. In gliomas, FGFR overexpression or gene fusion events, notably the FGFR3-tyransforming acidic crimp 3 (TACC3) fusion, can aberrantly activate these downstream pathways, promoting tumor cells proliferation, migration, and invasion (Singh et al., 2012). The resulting overactivation of FGFR3 is a recognized driver of tumor aggressiveness and resistance to therapy, establishing it as a compelling therapeutic target (Singh et al., 2012; Métais et al., 2023). It is important to note, however, that FGFR alterations are relatively uncommon in gliomas, especially in IDH-wildtype GBMs, accounting for less than 5% of cases (McDonald et al., 2022; Di Stefano et al., 2015). This low frequency may be eclipsed by the more pervasive influence of other pathways, such as EGFR and TP53, in this subset (Lamarca et al., 2020; Chan et al., 2022). Nevertheless, for the subset of patients with specific FGFR alterations like gene amplifications or fusions, targeting this pathway offers a highly relevant precision medicine strategy, with several FGFR inhibitors currently advancing through clinical trials (Métais et al., 2023). This review therefore focuses on the therapeutic potential of targeted therapies, specifically investigating the role of FGFR inhibitors in the management of gliomas. (Details of the literature search and evaluation are provided in the Supplementary Material).

**TABLE 1:** Common genetic mutations in gliomas and their effect.

Gene	Mutation type	Functional impact	Clinical significance	References
IDH1/2	Point mutation	Affects cell metabolism and inhibits tumour suppressor genes	IDH-mutated gliomas have a better prognosis and improved immune microenvironment	Miller et al. (2023), Pirozzi and Yan (2021)
TP53	Point mutation	Inhibits cell cycle regulation and increases DNA damage	Causing tumour cell proliferation and genetic instability	Cancer Genome Atlas Research Network et al. (2015), Joerger et al. (2025)
PTEN	Deletion/mutation	Inhibition of the PI3K/AKT pathway	Promoting tumour proliferation and invasion	Chen et al. (2019), Worby and Dixon (2014)
EGFR	Overexpression/amplification	Activate the MAPK pathway to enhance cell proliferation and survival	Associated with the development of malignant tumours and drug resistance	Freed et al. (2017), Habibi et al. (2025)
FGFR	Point mutations, gene amplifications, gene fusions, insertions, and deletions	Aberrant cell proliferation, migration, and survival, contributing to tumor growth, metastasis, and chemoresistance	Diagnostic markers and targeted therapies (e.g., Erdafitinib)	Zhang et al. (2024), Katoh et al. (2024)

## 2 Therapeutic effects of FGFR antagonists in glioma

### 2.1 The molecular architecture and signal transduction of the FGFR family

The FGFR family has at least 28 different members, with the four high-affinity receptor tyrosine kinase subtypes (FGFR1-4) being the most critical, alongside a receptor that binds ligands but lacks the intracellular kinase domain (FGFR5/FGFRL1) (Babina and Turner, 2017; Sleeman et al., 2001; Zlinska et al., 2025). These receptors, predominantly found on epithelial and endothelial cells, mediate cellular responses by binding to FGFs. At least 22 different FGF ligands have been identified, all sharing a conserved 120 amino acid sequence (Turner and Grose, 2010; Ornitz and Itoh, 2001). FGFs serve as pivotal regulators of cell proliferation, differentiation, and migratory capabilities throughout embryonic development. In the adult context, these factors are further implicated in key roles related to tissue maintenance and repair, the process of wound closure, and, pathologically, tumor angiogenesis (Narumi, 2018).

All four FGFR subtypes (FGFR1–4) are built around a shared, tripartite structure. This design includes an extracellular domain for ligand recognition, a single transmembrane linker, and an intracellular region that notably houses the catalytic tyrosine kinase domain (Chen et al., 2023; Lemmon and Schlessinger, 2010). The ligand-binding extracellular region is structured from three immunoglobulin (Ig)-like modules, conventionally labeled IgI, IgII, and IgIII (Zhuang et al., 2020; Chellaiah et al., 1994). Specifically, IgII and IgIII are crucial for mediating the binding of FGF ligands and heparin sulfate proteoglycans (HSPGs), which collectively determine ligand specificity and receptor activation (Mohammadi et al., 2005; Ferguson et al., 2021). The binding of the FGF ligand facilitates the dimerization of two receptor monomers, which in turn induces the trans-autophosphorylation of the intracellular tyrosine kinase domain at multiple key sites (e.g., Y653/Y654). This phosphorylation event is essential for activating the receptor’s enzymatic activity and initiating signal transduction (Edman et al., 2024; Liu et al., 2019).

Among them, FRS2α (FGFR substrate 2α) is one of the most important adaptor proteins. Following ligand engagement, the receptor’s cytoplasmic domain undergoes phosphorylation, creating binding sites for adaptor proteins. This phosphorylation step specifically facilitates the recruitment of the GRB2-SOS complex, which, in turn, initiates the canonical RAS-RAF-MEK-ERK/MAPK signaling cascade. The ultimate result of this pathway activation is the promotion of cellular proliferation and differentiation (Ong et al., 2001; Clark and Soriano, 2023). At the same time, FRS2α can also interact with GAB1 and PI3K to activate the PI3K-AKT pathway, regulating cell survival and anti-apoptosis (Lee et al., 2024). Moreover, FGFR can directly phosphorylate PLCγ, mediating the activation of the nuclear factor-κB (NF-κB) (Wang et al., 2024).

Collectively, the FGFR family establishes a complex signaling network through ligand-dependent dimerization and cross-regulation of multiple pathways, positioning it as a central player in normal cell function and tumor development.

### 2.2 Aberrant FGFR signaling and pathological roles in glioma

Dysregulation of FGFR signaling is implicated in numerous diseases; for instance, specific point mutations in FGFR1, FGFR2, and FGFR3 are associated with several human skeletal dysplasias and craniosynostosis syndromes (Webster and Donoghue, 1997). In the context of the central nervous system (CNS), FGFR1 and FGFR3 are predominantly expressed in astrocytes, while FGFR2 expression is restricted to oligodendrocyte cell lines (Falasca et al., 1998). Aberrant expression and signaling of FGFRs are directly linked to glioma progression and prognosis.

In astrocytomas, FGFR1 protein expression rises with the WHO grade, and elevated FGFR1 expression in malignant gliomas is generally not attributed to gene amplification, but rather to alterations in the ratio of its splice isoforms (Yamaguchi et al., 1994). A shift in the relative abundance of the alternatively spliced FGFR1 α and β isoforms is observed during the development of malignant brain tumors, in parallel with upregulated FGFR1 gene transcription. Furthermore, FGFR1 signaling confers a radioresistant phenotype to glioma cells, utilizing the downstream effectors plc1γ and hif1α to drive this effect. (Gouazé-Andersson et al., 2016). Evidence indicates that potentiated FGF2-FGFR1 signaling within the tumor cells contributes to the malignant phenotype through two specific molecular routes: it stimulates cellular proliferation through the AKT/MAPK cascade and enhances motility through the RAC1/CDC42 system (Jimenez-Pascual and Siebzehnrubl, 2019). Taken together, FGFR1 serves as a critical modulator of malignant glioma pathology, orchestrating major functions that encompass accelerated growth, enhanced invasion, diminished sensitivity to treatment, and stem-like capability.

Conversely, in patients diagnosed with glioma, diminished expression of FGFR2 and its IIIb/IIIc splice variants shows a strong inverse correlation with tumor malignancy level, consequently predicting poorer patient survival rates (Ohashi et al., 2014). Although elevated FGFR2 expression is linked to reduced tumor cell proliferation, the exact relationship between FGFR2 signaling and cell-cycle regulation is not yet fully understood (Zeng et al., 2022; Guo et al., 2024). Gene ontology analysis for FGFR3 points to its involvement in cell differentiation. Single-cell RNA-Seq analysis recently performed on GBM specimens uncovered a sharp five-fold increase in FGFR3 transcript abundance within the invasive marginal cells compared to the core. This observation points to FGFR3’s involvement in invasion, but conclusive direct evidence has not yet been established (Darmanis et al., 2017). Significantly, as the astrocytoma grade progresses, FGFR4 abundance rises specifically at the protein level. This increase is uncoupled from its mRNA expression, implying control via translational or stability mechanisms (Schramm et al., 2019). Since various combinations of FGFRs and splice isoforms play distinct roles in mediating FGF signaling within GBM cells, a detailed single-cell analysis of cell-surface FGFR expression is essential to thoroughly understand receptor heterogeneity within tumors and lay a precise foundation for developing novel targeted blockade approaches in glioma treatment.

## 3 Progress of FGFR inhibitors in gliomas

FGFR inhibitors can inhibit the proliferation, migration and invasion of glioma cells by suppressing the kinase activity of FGFR and blocking the activation of downstream signaling pathways. Clinical investigations have compellingly shown that the co-administration of FGFR blockade with alternative targeted agents leads to a marked improvement in therapeutic results. This strategy often relies on synergistic anti-tumor effects to achieve better patient outcomes.

### 3.1 Identification of FGFR signaling as a therapeutic vulnerability in glioma

Since the discovery of the first FGF in 2001, research related studies in various tumours have been increasing, and for gliomas FGFR has been screened as a new therapeutic target. A notable case study involves diffuse intrinsic pontine gliomas (DIPGs), which are highly aggressive brain tumors in children that develop in the pons. These tumors are marked by an infiltrative growth pattern and a poor prognosis, with a median overall survival of just 8–11 months (Mackay et al., 2017; Hargrave et al., 2006) DIPGs are commonly characterized by the recurring K27M mutation in the histone H3 genes (H3F3A and HIST1H3A/B/C). To uncover potential therapeutic targets in this difficult-to-treat cancer, large-scale knockout strategies using pooled short hairpin (sh) RNA libraries, in combination with next-generation sequencing, have been applied (Schramm et al., 2019). The implementation of this refined screening technology led to the simultaneous identification of PP2A and FGFR signaling as actionable targets in DIPG. Critically, this finding lends further support to the therapeutic hypothesis of inhibiting FGFRs family members across the spectrum of high-grade gliomas.

### 3.2 Mechanisms of FGF/FGFR-driven oncogenicity in glioma

Based on published data, FGFR is implicated in governing key malignant behaviors of glioma cells, specifically cell growth, invasion, and evasion of apoptosis, through a network of varied signaling pathways. We will therefore proceed to provide an in-depth explanation of the FGF/FGFR axis’s mechanisms of action.

One fundamental mechanism involves the regulation of cancer stem cell characteristics. Studies using C6 glioma cells demonstrated that FGF-2 is a potent inducer of Nestin expression. Nestin, a type VI intermediate filament protein, is commonly employed as a marker for neurogenic and myogenic precursor cells, and is linked to tumor stemness and malignancy. The FGF-2-induced Nestin expression is primarily mediated through FGFR1/3 and the subsequent activation of the Ras-Raf-ERK pathway, which culminates in the activation of the transcription factor Sp1 (Chang et al., 2013). This highlights FGFR signaling’s critical role in maintaining the malignant phenotype of glioma cells.

The FGFR signaling cascade is instrumental in driving the migratory and proliferative capabilities of glioma cells, partially through its association with cell adhesion molecules. Specifically, the L1CAM (also designated L1 or CD171), a transmembrane glycoprotein belonging to the Ig superfamily, has been documented to engage with FGFR family members through its outer cellular domain (Szele et al., 1994; Schmid and Maness, 2008). Research has demonstrated that L1CAM enhances glioma cell motility and proliferation by activating FGFR. Notably, silencing L1 expression and/or inhibiting FGFR activity *in vitro* leads to the complete cessation of cell migration. This mechanism confirms that the soluble L1 extracellular domain influences glioma cells through FGFRs, promoting their motility and proliferation. Effective treatment modalities for aggressive gliomas are likely contingent upon the concurrent disruption of L1, FGFRs, and integrin receptors. Such an approach is essential to significantly impair key oncogenic outputs, notably cell survival and proliferative capacity (Mohanan et al., 2013).

The preclinical utility of FGFR inhibition has been validated with various agents. AL3810, a molecular dual inhibitor of VEGFR and FGFR, is one such compound that has advanced to Phase II clinical trials in oncology. However, its application in glioma is severely limited by poor brain penetration due to its lack of interaction with the extracellular region and insufficient recognition of the blood-lymphatic barrier (BBTB). This underscores a major challenge for all small-molecule FGFR inhibitors targeting CNS malignancies. To overcome this, innovative strategies are necessary, such as the proposed use of targeted, AL3810-modified manganese liposomes with affinity for α_*v*_β_3_ integrins to enhance targeting ability and improve brain penetration (Li et al., 2021).

Interaction with other growth factor pathways contributes to the enhanced complexity of cellular oncogenic cues, notably involving the FGF/FGFR axis. Illustratively, transforming growth factor-β (TGF-β) signaling can selectively adjust the function of the pro-invasive basic FGF this modulation varies based on the specific cellular milieu. When bFGF levels are low, the non-canonical TGF-β pathway inhibits FGFR signaling by deactivating FGFR Substrate 2 (FRS2), leading to a non-motigenic phenotype. Conversely, in the presence of high bFGF, the bFGF-induced negative feedback regulation of FRS2 by ERK1/2 is overridden, leading TGF-β to inhibit FRS2 inactivation and restore pro-migratory signaling. This complex interaction highlights FRS2 as a key signaling hub, coordinating bFGF and TGF-β signals to regulate tumor cell invasion. Consequently, targeting FRS2 has emerged as a promising strategy to disrupt abnormal FGFR signaling by interfering with this essential convergence point (Santhana Kumar et al., 2018).

In summary, the confluence of high-throughput screening data, detailed mechanistic studies involving the promotion of stemness (via Nestin) and motility (via L1CAM), and the discovery of crucial signaling crosstalk and regulatory loops (via FRS2 and TGF-β). Collectively, these data rigorously validate the FGFR pathway as a highly significant and indispensable target for therapeutic intervention against gliomas. Future success hinges on developing compounds with superior blood-brain barrier penetrance and exploiting combination strategies that overcome the pathway redundancy central to glioma malignancy.

### 3.3 Diverse mechanisms of aberrant FGFR activation

Aberrant activation of the FGFR signaling axis in gliomas occurs through a diverse spectrum of molecular mechanisms, including point mutations, gene amplification, alternative splicing variants, and crucial gene fusions, each conferring a distinct oncogenic potential. Recurrent FGFR1 mutations (e.g., N546K, K656E) (Capone et al., 2022; Rivera et al., 2016; Jain et al., 2024) and Kinase Domain Duplications (KDDs) are predominantly observed in pediatric-type low-grade gliomas, leading to constitutive receptor activation and strong induction of the downstream MAPK/ERK pathways (Chen M. et al., 2024; Pathak et al., 2017). Furthermore, while less frequent, FGFR1 and FGFR3 amplifications occur in adult high-grade gliomas, contributing to receptor overexpression and ligand-independent signaling. Alternative splicing variants of FGFR1 and FGFR3 may also enhance ligand affinity and signal output, further driving glioma cell proliferation and invasion. Mong all structural alterations, oncogenic FGFR–TACC fusions represent the most characteristic and well-studied subtype-specific events in gliomas. These transcript fusions, caused by chromosomal rearrangements, represent a well-established category of somatic mutations that produce powerful, druggable oncoproteins, similar to the landmark discovery of BCR-ABL1 in chronic myeloid leukemia (Mitelman et al., 2007; Wu et al., 2013). Kinase fusions involving the FGFR and NTRK families are now commonly detected across various tumor types, including glioblastoma (4.4%) and low-grade gliomas (1.5%) (Singh et al., 2012; Shaw et al., 2013).

### 3.4 FGFR-TACC fusions: a defining oncogenic event

This research analyzed the full range of FGFR genetic changes detected in 101 cases of WHO grade IV diffuse gliomas, successfully bringing to light several novel FGFR2 and FGFR3 fusions. Notably, FGFR2 fusions were identified in IDH-mutant glioblastomas, a tumor type where the presence of oncogenic FGFR alterations was previously unrecognized (Lasorella et al., 2017). Large-scale genomic profiling has established that the FGFR3–TACC3 fusion is the most frequent FGFR rearrangement, recurring in approximately 3%–4% of IDH-wild-type GBM, where it often serves as the dominant driver alteration (Di Stefano et al., 2015). Other, rarer fusions have also been identified, such as FGFR1-TACC1 (Perwein et al., 2021; Bale, 2020) and FGFR2-CTNNA3 (Zhao et al., 2022) fusions are rare events predominantly found in IDH-mutant lower-grade gliomas (≤1%). Beyond these canonical TACC partners, next-generation sequencing has revealed a spectrum of noncanonical fusions (e.g., FGFR3-FASN, FGFR3-BAIAP2L1) in both primary and recurrent gliomas (Diaz et al., 2024; Gu et al., 2021). These diverse fusion variants can alter receptor localization, dimerization, and downstream adaptor interactions (like FRS2 and GRB2), thereby diversifying the oncogenic signaling output via the MAPK/ERK, PI3K/AKT, and JAK/STAT cascades (Ornitz and Itoh, 2015; Katoh, 2016).

**FIGURE 1:**
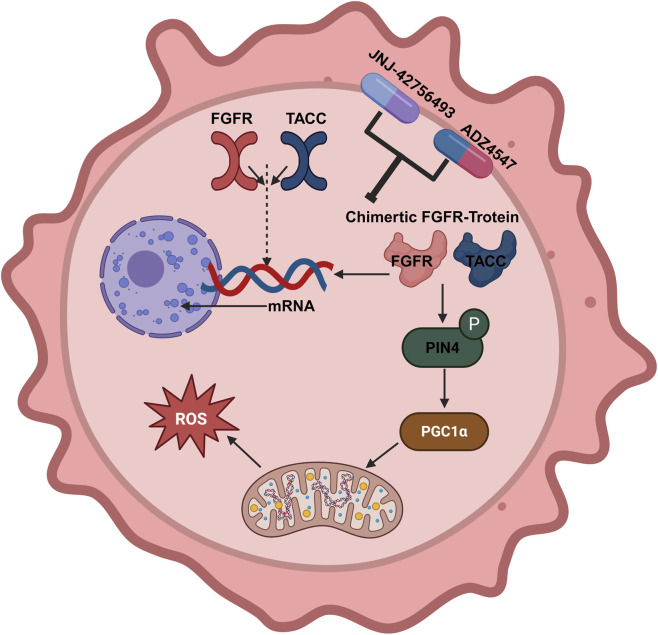
The mechanism of FGFR3-TACC3 fusion protein in glioma occurrence.

The discovery of FGFR-TACC fusions in GBM has generated significant enthusiasm, suggesting that FGFR kinase inhibition could be a viable therapeutic option for this highly aggressive disease. Studies have shown that FGFR3-TACC3 positive tumors are aggressive, fast-growing high-grade gliomas. Primary brain tumors frequently exhibit deregulation of FGFR1 via mechanisms like gain-of-function mutations, kinase domain amplification, and translocation, predominantly with the partner gene TACC1. Separate from this, FGFR2 fusions with CTNNA3, KIAA1598/SHTN1, or VPS35 (Zanazzi et al., 2020), as well as FGFR3-TACC3 fusions (Singh et al., 2012), are also observed. Mechanistically, the FGFR-TACC fusion is implicated in initiating chromosomal instability (CIN), with its growth-promoting function complementing the loss of mitotic fidelity and aneuploidy to drive full-blown tumor formation (Singh et al., 2012) (Figure 1).

Crucially, preclinical evidence has demonstrated the therapeutic efficacy of FGFR kinase inhibition in gliomas carrying the FGFR-TACC rearrangement. A specific FGFR inhibitor, JNJ-42756493, exhibited tolerable toxicity and significant anti-tumor activity in preclinical models of FGFR3-TACC3-positive gliomas, providing preliminary evidence of sensitivity for clinical trials (Di Stefano et al., 2015). Alterations in FGFR1-4 are widespread in various types of solid tumors, with most of them involving gene amplification and mutations. FGFR1-4 fusions occur only in a small number of cancer cases. Patients with glioblastoma carrying the FGFR3-TACC3 fusion may benefit from anlotinib (Gu et al., 2021). This suggests that low-prevalence, dominant driver lesions like the FGFR-TACC fusions may define a specific niche for this class of targeted drugs.

### 3.5 Distinct metabolic vulnerabilities and clinical implications

Further intensifying the interest in this alteration, FGFR3-TACC3 fusion has been linked to a unique metabolic phenotype characterized by the activation of mitochondrial function (Frattini et al., 2018). Tumors with the F3-T3 fusion show activation of oxidative phosphorylation and mitochondrial biosynthesis, rendering them unexpectedly sensitive to inhibitors of oxidative metabolism. This metabolic reliance is linked to the F3-T3-PIN4 pathway, which activates peroxisome biogenesis and the synthesis of new proteins (Frattini et al., 2018). This convergence on PGC1α via intracellular Reactive Oxygen Species (ROS) promotes mitochondrial respiration and tumor growth, revealing that dependence on mitochondrial metabolism is a potential therapeutic vulnerability for F3-T3-carrying GBMs.

For clinical translation, FGFR3 immunohistochemistry (IHC) has been established as a useful screening tool for detecting FGFR3 alterations and can be incorporated into the diagnostic workflow for IDH-wildtype gliomas (Lasorella et al., 2017; Schittenhelm et al., 2021). The detection of FGFR3 overexpression can then guide the selection of samples for confirmatory NGS-based diagnostic tools to identify specific fusions and sequence variants (Perwein et al., 2021). Over the past decade, substantial strides have been made in synthesizing FGFR inhibitors characterized by heightened potency and specificity, with several compounds, including ADZ4547, currently in clinical trials for cancer, generating cautious optimism for future research. Importantly, the biological behavior, metabolic phenotype, and ultimately, the drug sensitivity of gliomas appear to vary with the specific fusion partners (e.g., TACC3 vs. FASN), underscoring the necessity of comprehensive genomic profiling at both diagnosis and recurrence to optimize FGFR-targeted strategies.

### 3.6 Rationale and efficacy of FGFR inhibitors in glioma therapy

**FIGURE 2:**
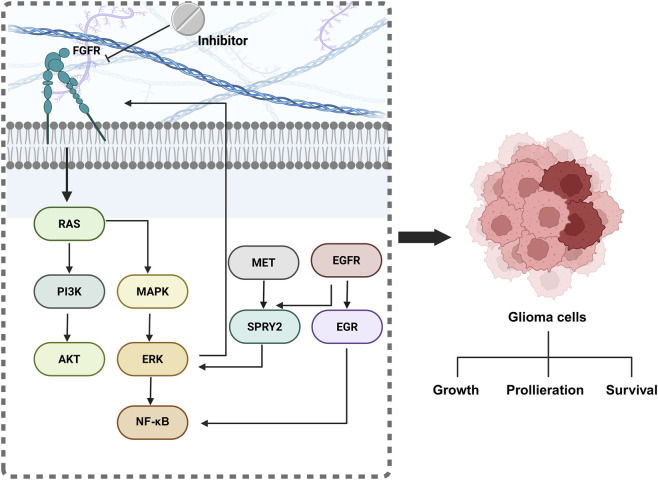
Mechanism of action of FGFR inhibitors.

The FGFR amily of receptors is indispensable for governing fundamental cellular processes such as growth, survival, and motility. Importantly, aberrant FGFR signaling has been mechanistically linked to the onset and progression of numerous malignancies, including glioblastomas and other gliomas (Schuler, 2020; Xie et al., 2020; Dong et al., 2023). FGFR inhibitors have become a promising treatment approach for glioma, a type of brain tumor that is notoriously difficult to treat (Figure 2). In gliomas, the aberrant activation of FGFR signaling drives uncontrolled tumor growth, invasion, and promotes resistance to conventional therapies. By specifically targeting FGFRs, these small-molecule inhibitors effectively block crucial downstream cascades, primarily the PI3K/Akt and MAPK pathways, which are essential for tumor progression (Table 2) (Clark and Soriano, 2024; Katoh and Nakagama, 2014). Preclinical models consistently demonstrate that FGFR inhibition can suppress glioma cell proliferation, induce apoptosis, and significantly reduce tumor invasiveness (Katoh and Nakagama, 2014). Furthermore, this targeted approach shows potential in overcoming resistance to standard treatments, including chemotherapy and radiation (Patel et al., 2020; Fisher et al., 2020).

**TABLE 2:** Effects of FGFR inhibitors and progress in clinical research.

Drug name	FGFR target	Main mechanism	Clinical trial phase	Clinical efficacy	References
Erdafitinib	FGFR1-4	Inhibits FGFR kinase activity and blocks downstream signaling pathways	Phase III clinical trial	Effective in patients with FGFR mutations	Zisi et al. (2023), Cabezas-Camarero et al. (2024), Padovan et al. (2023)
AZD4547	FGFR1-3	Targeting FGFR receptors, inhibiting PI3K/AKT and MAPK signaling pathways	Phase II clinical trial	Demonstrated good safety and efficacy	Picca et al. (2024), Holzhauser et al. (2020)
Infigratinib (BGJ398)	FGFR1-3	Inhibits FGFR kinase, reducing tumor cell proliferation and invasion	Clinical Phase I/II	Showing promise in glioma treatment	Lassman et al. (2022), Chen et al. (2024b)
PD173074	FGFR tyrosine kinase domain	Targeting FGFR receptors, inhibiting PI3K/AKT and MAPK signaling pathways	Preclinical studies	A promising therapeutic strategy for glioblastoma	Anderson and Galileo (2016)
Regorafenib	VEGFR1–3, TIE2, KIT, RET, RAF1/BRAF	RAS/MAPK, PI3K/Akt/mTOR and Fos/Jun pathways	Phase II clinical trial	Modest survival benefit in recurrent glioblastoma patients	Subr et al. (2020)

The therapeutic promise of FGFR inhibitors is currently being rigorously assessed, but their ultimate effectiveness in treating gliomas is contingent upon overcoming challenges such as tumor heterogeneity and minimizing potential off-target effects. Despite these hurdles, FGFR inhibitors represent a novel and exciting approach to glioma treatment, offering hope for more targeted and effective therapies. RTKs, such as FGFRs, are receptors located on the cell surface that regulate key signaling pathways, including the Ras/MAPK/ERK and Ras/PI3K/AKT cascades (Regad, 2015). Studies have demonstrated a synergistic anti-glioma effect when FGFR inhibitors are combined with agents that modulate the Cdc2-like kinase (CLK) family. RTKs, including FGFRs, are cell surface receptors that control important signaling pathways, such as the Ras/MAPK/ERK and Ras/PI3K/AKT cascades. In glioblastoma models, the combined inhibition of FGFR and reduced CLK2 expression synergistically induced apoptosis and cell cycle arrest, suggesting that pathways regulated by CLK2 may mediate resistance to single-agent FGFR inhibition (Park et al., 2020). Given the central role of these pathways in tumor progression, preclinical and clinical studies are actively exploring the safety and efficacy of FGFR inhibitors, with a significant emphasis on their integration into rational combination therapies to improve patient outcomes.

Inhibition of ribosome biogenesis (RiBi) perturbation triggers the impaired ribosome biogenesis checkpoint (IRBC), leading to cell cycle arrest and death (Zisi et al., 2022). BMH-21 is a small-molecule inhibitor that targets RNA polymerase I transcription (Peltonen et al., 2014), was found to synergistically inhibit glioma cell growth when combined with the FGFR inhibitor Erdafitinib (Zisi et al., 2023). This supports a rational combination approach that simultaneously targets receptor signaling and fundamental cellular machinery. Clinical evaluation is providing initial validation for this targeted approach.Infigratinib, a specific FGFR inhibitor, has been assessed in a multicenter Phase II trial with patients who have recurrent gliomas containing FGFR mutations or amplifications (Lassman et al., 2022; Chen Y. et al., 2024). The cell adhesion molecule L1CAM promotes glioblastoma cell motility and proliferation. Researchers found that inhibiting FGFR (using agents like PD173074), integrins, or FAK effectively reduced L1CAM-stimulated glioblastoma cell movement and growth. This highlights the potential of using FGFR inhibitors in combination with integrin and FAK inhibitors to specifically target the migration and invasive phenotype critical to glioma progression (Anderson and Galileo, 2016). While the overall response rate was modest, the drug demonstrated measurable anti-tumor activity, particularly within the molecularly defined subset of patients with FGFR alterations. The treatment was generally well-tolerated, with fatigue and rash being common side effects, indicating that Infigratinib may provide a promising therapeutic option for FGFR-driven recurrent gliomas.

Regorafenib is a multi-targeted, oral tyrosine kinase inhibitor that exerts inhibitory effects against several cancer-driving pathways. Its targets encompass angiogenic receptors (VEGFR1-3, TIE2), key oncoproteins (KIT, RET, RAF1/BRAF), and other RTKs, with its activity against the FGFR family of receptors being characterized by variable potency levels (Mross et al., 2012; Strumberg and Schultheis, 2012). In recurrent glioblastoma, randomized Phase II data from the REGOMA trial reported a modest but statistically significant improvement in overall survival compared to lomustine in the second-line setting, cementing Regorafenib as a clinically relevant option for selected patients. This success is likely attributable to the drug’s broad anti-angiogenic and anti-proliferative profile, which addresses the complex microenvironmental drivers of recurrent disease, rather than highly selective FGFR blockade alone. Despite this, the clinical use of multi-kinase inhibitors is complicated by trial heterogeneity, toxicity management (e.g., hand–foot reaction, hypertension), and the critical need to define predictive biomarkers (Mross et al., 2012; Strumberg and Schultheis, 2012).

Enhancing CNS exposure of FGFR inhibitors requires optimizing key pharmacokinetic factors that influence blood-brain barrier (BBB) penetration (Langen et al., 2019). Critical determinants include “molecular weight (<450 Da)”, “moderate lipophilicity (logP 2-4)”, “low polar surface area”, and “minimal interaction with efflux transporters” such as P-gp and BCRP. Structural optimization strategies-such as reducing polarity, masking hydrogen-bond donors, and avoiding cationic centers-can decrease transporter recognition and improve passive diffusion (Nau et al., 2010). For new-generation agents like “futibatinib (TAS-120)”, covalent binding offers sustained target inhibition, while “liposomal or nanoparticle formulations of erdafitinib” can enhance BBB penetration, prolong systemic circulation, and exploit tumor vasculature permeability (Goyal et al., 2023; Sootome et al., 2020). Surface modification with ligands targeting transferrin or insulin receptors may further facilitate receptor-mediated transcytosis. Additionally, “convection-enhanced delivery (CED)” or “focused ultrasound (FUS)” can transiently disrupt the BBB to improve local drug concentration (Mehta et al., 2017; Alekou et al., 2021). Future designs should integrate medicinal chemistry optimization with nanocarrier systems and localized delivery to achieve sufficient unbound brain drug levels, enabling FGFR inhibitors to effectively target glioma within the CNS.

### 3.7 Blocking the effects of FGFR inhibitors on other pathways

Evan K. Day and colleagues discovered that sprouty2 (SPRY2), typically regarded as a tumor suppressor in certain cancers, actually facilitates tumor growth and resistance to receptor tyrosine kinase inhibitors in glioblastoma. In this study, we uncover SPRY2-dependent bypass signaling mechanisms in glioblastoma that contribute to resistance against EGFR and MET (hepatocyte growth factor receptor) inhibition (Day et al., 2020). The intricate molecular landscape of GBM resistance to EGFR and MET inhibitors is defined by a SPRY2-mediated ERK eactivation loop. Initially, treatment suppresses SPRY2 expression; however, the subsequent activation of the NF-κB pathway induces autocrine FGFR signaling, which in turn reinstates ERK activity and restores SPRY2 transcription. ThisFGFR--driven bypass mechanism fundamentally dictates acquired therapeutic resistance. Functionally, cell death induction by EGFR/MET blockade is significantly greater when this FGFR -mediated ERK rebound is absent, or when SPRY2 is experimentally inhibited despite ERK reactivation. Importantly, studies utilizing specific ERK-bioluminescent reporter tumor explants confirmed this bypass operates *in vivo*. The data collectively suggest that simultaneous inhibition of FGFR presents a highly promising strategy to overcome this resistance and improve glioma treatment efficacy.

### 3.8 Advances in FGFR receptor mutations in glioma treatment

FGFR1 mutations, believed to originate in the subventricular zone, have been identified in midline pilocytic astrocytomas affecting the thalamus or brainstem (Jones et al., 2013). Spontaneous haemorrhage has been reported in up to 24% of patients with mucinous astrocytoma (Schramm et al., 2019; Mackay et al., 2017; Hargrave et al., 2006). Recent research has indicated that FGFR1 mutations are linked to spontaneous hemorrhage in pediatric low-grade gliomas (Ishi et al., 2021), although the exact mechanism remains unclear and may be driven by FGFR1’s direct effects rather than the MAPK pathway (Karthigeyan et al., 2019; Linscott et al., 2008). In tumors from patients studied by Fomchenko et al. (2021), the mutant allele frequencies of FGFR1p.K656E and FGFR1p.V561M were found to be similar, suggesting that these mutations may arise at comparable time points. The data imply a functional diversity among mutations, where the activating mutation type determines the specific downstream signaling pathways that are preferentially engaged. For example, mutations may alter the secondary junction protein’s affinity, triggering the PI3K and MAPK pathways, or enable preferential activation of the STAT and PLCγ pathways. This leads to a dose-dependent effect, and it is experimentally known that targeting the PI3K pathway in combination with FGFR1 inhibition is a potential strategy for treatment.

## 4 Discussion and future perspectives

The research into the origin, molecular classification, and therapeutic targeting of gliomas is evolving rapidly, driven by continuous molecular updates that refine our understanding of GBM and its treatment (Verdugo et al., 2022). Beyond well-established genetic and radiation-related risk factors, multi-omic profiling continues to uncover novel molecular determinants that drive gliomagenesis and therapeutic resistance. The FGFR signaling axis has emerged as a key regulator of tumor proliferation, stemness maintenance, and metabolic adaptation. This review provides an updated and integrative overview of FGFR dysregulation across mutation, amplification, fusion, and splicing variants, systematically distinguishing their distribution among IDH-WT and IDH-mutant glioma subtypes. Critically, the identification of these targetable alterations, particularly novel FGFR2 and FGFR3 fusions, is underpinned by recent findings demonstrating their association with an aggressive phenotype and distinct gene expression programs in GBM (Georgescu et al., 2021). This evidence reinforces the clinical and mechanistic implications of noncanonical FGFR fusions (e.g., FGFR1-TACC1, FGFR2-CTNNA3, and FGFR3-TACC3) and underscores their potential as molecular biomarkers for patient stratification and therapeutic response prediction. Furthermore, we discussed emerging pharmacological strategies to enhance CNS exposure of FGFR inhibitors, including the development of liposomal formulations and highly BBB-permeable agents such as futibatinib, providing a refined framework for rational drug design in FGFR-driven gliomas.

The path forward for FGFR-targeted therapies must align with this continuous molecular refinement. Future research should focus on several key directions to translate molecular understanding into durable clinical benefit. First, precision patient selection based on next-generation sequencing at both diagnosis and recurrence will be essential to accurately identify the actionable and aggressive FGFR alterations. Second, improving drug pharmacokinetics and blood-brain barrier penetration—through nanocarriers, lipid conjugation, or P-gp/BCRP efflux modulation—remains critical to achieve effective CNS exposure. Third, combination strategies integrating FGFR inhibition with agents like the multi-kinase inhibitor Regorafenib, immune checkpoint blockade, or DNA damage response modulators may overcome molecular resistance and enhance therapeutic synergy. Finally, incorporating real-time molecular monitoring and liquid biopsy-based detection of FGFR fusions is necessary for adaptive treatment optimization. Altogether, the evolving landscape of molecular biology and targeted therapy holds great promise for transforming the management of FGFR-driven gliomas.

## Glossary

BBB: Blood-brain barrier
BBTB: Blood-lymphatic barrier
CED: Convection-enhanced delivery
CIN: Chromosomal instability
CLK: Cdc2-like kinase
CNS: Central nervous system
Dipgs: Diffuse pontine gliomas
EGFR: Epidermal growth factor receptor
EMS: Eloproliferative syndrome
FGFR: Fibroblast growth factor receptor
Fn: Fibronectin
FUS: Focused ultrasound
GBM: Glioblastoma
GSC: Glioma stem cell
HSPGs: Heparin-like glycosaminoglycans
IDH: Isocitrate dehydrogenase
IDH-WT: IDH-wildtype
IF: Intermediate filament
Ig: Immunoglobulin
IHC: Immunohistochemistry
IRBC: Impaired ribosome biogenesis checkpoint
L1LE: L1 extracellular region
NF-κB: Nuclear factor-κB
RiBi: Ribosome biogenesis
ROS: Reactive oxygen species
RTKs: Receptor tyrosine kinases
Sh: Short hairpin
SPRY2: Sprouty2
TACC: Transforming acidic crimp
TKS: Tyrosine kinase
VEGFR: Vascular endothelial growth factor receptor
