# Correction: Fibroblast growth factor receptor inhibitors in glioma: a narrative review of recent advances

**DOI:** 10.3389/fphar.2026.1811017

**Published:** 2026-02-24

**Authors:** Mingyu Han, Zhaokai Zhou, Bi Qian, Yuanqi Zhang, Cheng Peng, Fu Peng

**Affiliations:** 1 West China School of Pharmacy, Sichuan University, Chengdu, China; 2 Key Laboratory of Standardization of Chinese Medicine (Chengdu University of Traditional Chinese Medicine), Ministry of Education, Chengdu, China; 3 Department of Urology, The Second Xiangya Hospital of Central South University, Changsha, Hunan, China; 4 Department of Radiation Oncology, Peking Union Medical College Hospital, Chinese Academy of Medical Sciences and Peking Union Medical College, Beijing, China; 5 Affiliated Hospital of Guangdong Medical University, Guangdong Medical University, Guangzhou, China; 6 Key Laboratory of Drug-Targeting and Drug Delivery System of the Education Ministry, Sichuan Engineering Laboratory for Plant-Sourced Drug and Sichuan Research Center for Drug Precision Industrial Technology, Sichuan University, Chengdu, China

**Keywords:** glioma, fibroblast growth factor receptor (FGFR), pathways, gene fusion, inhibitors

Author Yuanqi Zhang was erroneously spelled as Qiyuan Zhang.

The original article has been updated.

