# Supplementary material for: Fibroblast growth factor receptor inhibitors in glioma: a narrative review of recent advances
Source: Front Pharmacol. 2026 Jan 6;16:1714696. doi: 10.3389/fphar.2025.1714696 (PMC12816223; doi:10.3389/fphar.2025.1714696)
Supplement: Supplementary file 1 [file DataSheet1.docx]

**Supplement Materials**

**Materials and Methods**

**Search strategy**

A comprehensive and systematic literature search will be conducted across major electronic databases, including PubMed/MEDLINE, Embase, the Cochrane Central Register of Controlled Trials (CENTRAL), and Web of Science. The search strategy will employ a combination of Medical Subject Headings (MeSH terms) and free-text keywords related to the target, the intervention, and the disease. Key terms will encompass "Fibroblast Growth Factor Receptor," "FGFR," and specific inhibitors such as "Infigratinib," "Erdafitinib," "Regorafenib," "AZD4547," or "FGFR inhibitor," combined using Boolean operators (AND/OR) with disease terms like "Glioma," "Glioblastoma," "GBM," "Astrocytoma," and "Diffuse Intrinsic Pontine Glioma." The search will be restricted to the English language and span from the inception of each database to the current date. Furthermore, we will manually screen the reference lists of all included articles, relevant systematic reviews, and search clinical trial registries (e.g., ClinicalTrials.gov) to ensure maximum inclusivity of relevant data.

**Inclusion criteria**

Studies will be included in the Meta-Analysis based on the following PICO (Population, Intervention, Comparator, Outcome) principles. The Population must comprise patients diagnosed with glioma (any WHO grade) who are receiving treatment, with a preference for studies reporting on patients with known FGFR molecular alterations (e.g., mutations, amplifications, FGFR-TACC fusions). The Intervention must involve treatment with an FGFR inhibitor, either as a monotherapy or as part of a combination regimen. The Comparator group must consist of patients receiving standard-of-care treatment, best supportive care, or placebo. The Outcome must report at least one quantifiable efficacy or survival metric, such as Overall Survival (OS), Progression-Free Survival (PFS), or Objective Response Rate (ORR). Only Randomized Controlled Trials (RCTs) or high-quality comparative prospective or retrospective clinical cohort studies reporting control group data will be eligible for inclusion.

**Exclusion criteria**

Non-clinical studies, including all in vitro, cell line, or animal model experiments, will be excluded. Case reports, case series, letters, conference abstracts without subsequent full-text publications, narrative reviews, and expert opinions will also be excluded. Any clinical study lacking specific data on the efficacy outcomes (OS, PFS, or ORR) will be eliminated. Finally, studies where the primary disease population is not glioma, or studies that are determined to be redundant publications of the same patient cohort, will be excluded; in the latter case, only the most complete and recent report will be retained.

**Data extraction**

Two independent investigators will perform data extraction using a standardized, pre-designed data sheet, with discrepancies resolved through consensus or consultation with a third reviewer. Extracted data will encompass detailed study characteristics (first author, publication year, study design, phase, and follow-up duration) and patient characteristics (glioma WHO grade, specific molecular alteration status, treatment line, and baseline performance status). Detailed intervention data will include the name, dosage, and administration schedule of the FGFR inhibitor, noting whether it was used as monotherapy or in combination. Crucially, outcome data will be extracted for all primary and secondary endpoints, specifically including Hazard Ratios (HRs) and their 95% Confidence Intervals (CIs) for time-to-event outcomes (OS and PFS), and raw event counts for dichotomous outcomes (ORR, Disease Control Rate, and Adverse Events) necessary to calculate Odds Ratios (ORs) or Risk Ratios (RRs).

**Outcome indicator analysis**

The primary outcome will be the summary estimate for OS and PFS, analyzed using the HR and its 95% CI. Secondary outcomes will include the ORR) analyzed using OR and 95% CI, and the incidence of Grade ≥ 3 Adverse Events (AEs), analyzed using the Risk Ratio (RR). Statistical pooling will be performed using standard methods in dedicated software (e.g., Review Manager or R). Heterogeneity across studies will be assessed using the *I*^2^ statistic and the Chi-square Q-test. If significant heterogeneity (*I*^2^ ≥ 50% or *P* ≤ 0.1) is detected, the Random-Effect model will be used; otherwise, the Fixed-Effect model will be applied. To explore sources of heterogeneity, subgroup analyses will be performed based on key factors such as the specific FGFR alteration type (e.g., fusion vs. mutation), the glioma type (e.g., DIPG vs. adult GBM), and the treatment type (monotherapy vs. combination therapy). Sensitivity analysis will also be conducted by sequentially excluding individual studies to test the robustness of the pooled results.

**Results**

The Results section will begin with a concise narrative describing the systematic search and selection process, visualized through a PRISMA flow diagram detailing the number of studies identified, screened, and ultimately included in the quantitative synthesis. This will be followed by a comprehensive table summarizing the characteristics of all included studies. The core findings will be presented using Forest Plots for all primary and secondary outcomes, clearly displaying the individual study estimates, heterogeneity statistics, and the pooled effect size (HR or OR). The narrative will specifically discuss the findings related to the primary outcomes (OS and PFS), highlighting the overall impact of FGFR inhibition. Finally, the results of the prespecified subgroup analyses will be presented and critically discussed, specifically addressing whether the efficacy of FGFR inhibitors is enriched in patients with certain molecular features (e.g., FGFR3-TACC3 fusion) compared to other subsets. The safety profile, including the pooled incidence of common Grade ≥ 3 AEs, will also be summarized. (*Supplement Table* S1).

***Supplement Table* 1. Characteriistics of included systematic**

| Anderson, H. J. (2016)*.*^107^ | Park, S. Y. *et al* (2020)*.*^101^ | Di Stefano, A. L. *et al* (2015)*.* ^81^ | Mross, K. *et al* (2012). ^101^ | Chen, Y. *et al* (2024). ^99^ | Lassman, A. B. *et al* (2022). ^98^ | **Author, year** |
| --- | --- | --- | --- | --- | --- | --- |
| Combination Strategy Study | Mechanistic Combination Study | Preclinical Validation/Follow-up | A phase I dose-escalation study | Efficacy and safety analysis | Multicenter Phase II Study | **Study type** |
| Glioblastoma (Cell line model) | Glioblastoma (GSC model) | GBM and Grade II-III Gliomas | Advanced solid tumors (renal cell carcinoma, colorectal carcinoma, osteosarcoma and ) | Adult glioma patients with tyrosine kinase alterations | Recurrent Gliomas (Harboring FGFR alterations) | **Cancer type** |
| Preclinical (*In vitro*) | Preclinical (*In vitro*/Xenograft) | Preclinical/Sensitivity Testing | Phase I | Phase II | Phase II | **Trial Phase** |
| FGFR Inhibitor (PD173074) in combination with Integrin or FAK Inhibitors | FGFR Inhibitor in combination with CLK2 knockdown | JNJ-42756493 (Specific FGFR inhibitor) | Regorafenib (Multi-kinase inhibitors) | Infigratinib (FGFR inhibitor) | Infigratinib (FGFR inhibitor) | **Intervention** |
| Single agent inhibition | FGFR inhibitor or CLK2 knockdown alone | Not applicable (Drug sensitivity in models) | The patients were included in 8 different cohorts, each receiving a different dosage. | Single-arm design implied (No explicit comparator) | Single-arm design implied (No explicit comparator) | **Comparison** |
| Glioblastoma cells | Glioblastoma Stem Cells (GSCs) | FGFR3-TACC3 positive gliomas (Preclinical models) | A total of 53 patients were included in 8 cohorts, with dose levels ranging from 10 to 220 milligrams per day. | Patients with FGFR mutations/amplifications (N not specified) | Patients with FGFR mutations/amplifications (N not specified) | **Sample (experimental vs. Control group)** |
| Reduction in Cell motility and proliferation, Disruption of L1CAM-stimulated signaling | Apoptosis, Cell cycle arrest, Synergistic induction of death | Therapeutic efficacy, Significant anti-tumor activity, Tolerable toxicity (Preclinical evidence) | Anti-tumor activity, Toxicity (Hand–foot reaction, Hypertension and diarrhea) | Overall Survival (OS), Anti-tumor activity, Objective Response Rate (ORR) (Modest), Safety profile (Gastrointestinal, General/Systemic, Hemic and Lymphatic) | Anti-tumor activity, Objective Response Rate (ORR) (Modest), Safety profile (Fatigue, Rash, Liver enzyme elevation) | **Measurements** |
